# Morphologically and immunohistochemically undifferentiated gastric neoplasia in a patient with multiple metastatic malignant melanomas: a case report

**DOI:** 10.1186/1752-1947-2-134

**Published:** 2008-04-30

**Authors:** Federico Alghisi, Pietro Crispino, Andrea Cocco, Antonio G Richetta, Francesco Nardi, Paolo Paoluzi, Danilo Badiali

**Affiliations:** 1 Gastroenterology Unit, Department of Clinical Sciences, Policlinico Umberto I, University 'La Sapienza', Viale del Policlinico, 00161 Rome, Italy; 2 Department of Dermatology, University 'La Sapienza', Rome, Italy; 3 Department of Pathology, University 'La Sapienza', Rome, Italy

## Abstract

**Introduction:**

Malignant melanoma is a neoplasia which frequently involves the gastrointestinal tract (GIT). GIT metastases are difficult to diagnose because they often recur many years after treatment of the primary cutaneous lesion and also manifest clinically at an advanced stage of the neoplasia. Furthermore, GIT metastases can appear in various morphological forms, and therefore immunohistochemistry is often useful in distinguishing between a malignant melanoma and other malignancies.

**Case presentation:**

We report the case of a 60-year-old man with a multiple metastatic melanoma who underwent an upper endoscopy to clarify the possible involvement of the gastric wall with a mass localized in the upper abdomen involving the pancreas and various lymph nodes, which was previously described with computed tomography. Clinically, the patient reported a progressive loss of appetite, nausea and vomiting. The upper endoscopy and histological examination revealed a gastric location of an undifferentiated neoplasm with an absence of immunohistochemical characteristics referable to the skin malignant melanoma that was removed previously.

**Conclusion:**

The present case report shows the difficulty in diagnosing a metastatic melanoma in the GIT and therefore, it seems worthwhile to consider metastatic malignant melanoma in the differential diagnosis of undifferentiated neoplasia.

## Introduction

Melanoma is one of the most common neoplasia. The incidence of melanoma has increased in the last three decades; in the United States it was estimated as 5.7 cases per 100,000 people in 1973 and has increased dramatically to 14.3 cases per 100,000 people in 1998 [1]. Meanwhile, the overall survival rate has mildly improved: the 5-year survival rate was 80.0% in the 1970s and it achieved 88.8% by the end of the last century. This probably reflects an increased disease incidence, as well as earlier diagnosis of melanoma and better therapeutic options developed during the last few decades [1].

Melanoma originates most frequently in the skin. Other possible, but less-frequent, primary locations are intraocular, subungueal and mucosal sites. After treatment of the primary lesion, melanoma recidivates in about one-third of patients, involving almost every major organ and tissue. The most common sites of metastases are the skin, lung and brain. Metastases in the gastrointestinal tract (GIT) are not rare, however, they are less frequent than the above-mentioned sites and they usually manifest clinically at an advanced stage of the neoplasia. Diagnosis requires careful inspection of the mucosa for metastatic lesion detection and biopsy and the use of special immunohistochemical stains [2].

The overall median survival time in patients with metastatic melanoma is 7.5 months with a 5-year survival rate of 6%. Patients with GIT metastases have a median survival time of 12.5 months with a 5-year survival rate of 14%. Survival is strictly related to three independent variables: (i) the initial site of metastases (p < 0.0001); (ii) the interval between treatment of the primary lesion and onset of metastases, the disease-free interval (DFI) (p = 0.0001); and (iii) the stage of disease preceding distant metastases (p = 0.0001). To date the preferred treatment choice for GIT metastases remains surgery. Surgery improves the survival rate significantly, especially when the resection is considered complete following microscopic examination. The median survival after complete resection is 48.9 months, compared with 5.4 months after an incomplete resection [3]. Surgery is also recommended for palliative treatment of GIT metastases, with symptom relief reported in the range of 77% to 100% of patients, depending on the site and the reason for resection.

## Case presentation

We report the case of a 60-year-old man with multiple metastatic melanoma, who presented to our unit with vomiting and was later diagnosed with a gastric neoplasia with no histological and immunohistochemical characteristics referable to a malignant melanoma.

He underwent a surgical excision of a cutaneous lesion, localized on the left sub-costal region. The histological findings suggested a melanocytic melanoma with fused cells, of nodular type, exceeding the reticular layer of derma (pT4a), of Breslow thickness 8.3 mm and Clark level IV. Two months later the patient underwent an axillary lymph node dissection with no histological evidence of nodal metastases. Furthermore, he was treated with six cycles of chemotherapy with dacarbazine and IL-2. Four months after concluding chemotherapy the patient underwent a total-body computed tomography (CT) scan revealing three low-density lesions on the II, III and VIII segment of the liver, which remained uninvestigated. The CT scan also revealed one nodule (<1 cm) on the apical segment of the right lung and one sub-pleural nodule (1 cm) on the basal segment of the left lung, accompanied by a thickened contiguous pleura. Due to these findings, the patient underwent a thoracotomy, but the histological examination only revealed the presence of fibrotic tissue.

**Figure 1:**
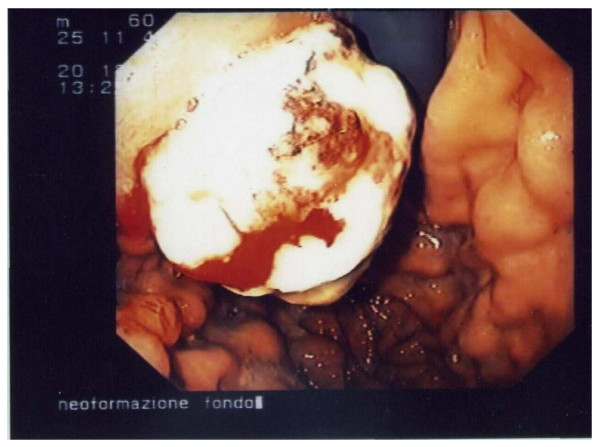
Endoscopic appearance of the gastric neoplasia.

**Figure 2:**
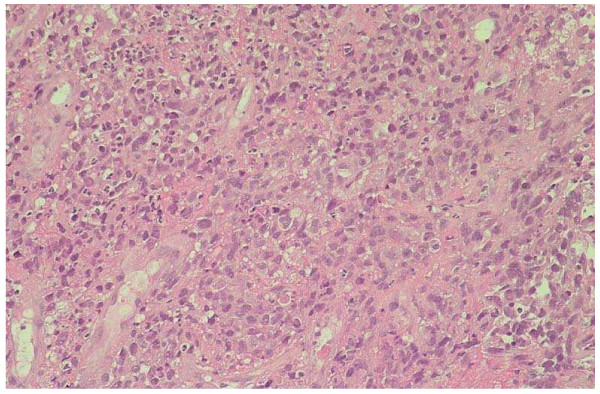
Histological pattern of the gastric neoplasia. Microscopic aspects reveal the presence of undifferentiated cells in which fused cells similar to the primitive melanoma can be distinguished.

During follow-up, the clinical condition of the patient remained stable for six months until the appearance of multiple masses localized on the left arm, both axillae and at the level of the primary melanoma surgical scar. The patient underwent a total-body bone scintigraphy that showed an increased concentration of the radioactive tracer (99mTc-MDP) in the left collarbone, II and VII right ribs, IV left rib, L2 and left acetabulum. A total-body CT scan showed multiple intra-peritoneal subcutaneous nodules, two metastatic lesions in the liver (IV and V segment), one in the spleen (1 cm) and one in the pancreas corpus (2 cm). A positron emission tomography (PET) scan confirmed the presence of multiple skeletal, muscular and nodal repetitive lesions. A treatment of three cycles of Interferon 5MU three times a week and Temozolomide 150 mg/m^2^/day 5 days a week for 4 weeks was started. However, the treatment was suspended after the second cycle due to side effects. The patient required admission to hospital due to his worsened clinical state, which included progressive asthenia, muscle and skeletal pain, nausea and vomiting. Five months after the last investigation the patient underwent a total-body CT again which confirmed the presence of multiple subcutaneous, muscular and nodal repetitive lesions; in addition, the CT scan revealed a gross lesion in the upper abdomen, probably due to confluent lymph nodes, undistinguishable from the gastric corpus and pancreas. Therefore, the patient was referred to our unit to evaluate the gastric wall involvement and its role in causing obstruction and vomiting. The patient underwent an upper endoscopy that showed a prominent mass at the passage between the fundus and the corpus of the stomach, with a hard consistency and largely covered by fibrin; the diameter of the lesion was about 3 cm (Figure 1). Histological findings suggested an undifferentiated neoplasia constituted prevalently by neoplastic fused cells (Figure 2). The immunohistochemical stains for melanoma (S100, tyrosinase, Melan A and HMB-45), carcinoma (CK), gastrointestinal stromal tumours (CD-34, vimentin and c-Kit) and lymphoma (LCA) were performed with negative results, except for a weak and focal expression of vimentin. No treatment for the gastric mass was started because of the patient's compromised clinical condition. Enteral nutrition was maintained until the patient's death, a month after the endoscopy.

## Discussion

Malignant melanoma is very likely to produce regional lymph node and distant metastasis. GIT metastases are frequent but rarely diagnosed. In fact, only 1% to 4% of GIT metastases are clinically diagnosed *ante mortem *in patients affected by malignant melanoma, while the frequency of GIT metastases is more than 60% in autopsy series. Moreover, melanoma is the most common metastatic tumour to the GIT; autopsy studies reported that 23% of GIT metastases derived from malignant melanoma. These data suggest that GIT metastases are difficult to diagnose, probably because symptoms are often absent or non-specific. Moreover, symptoms may be due to or modified by treatment of the primary tumour, such as chemotherapy or radiotherapy [3]. Patients with GIT metastases are usually investigated when they present with anaemia, gross bleeding, obstruction, abdominal pain or weight loss; these symptoms often arise in an advanced stage of the disease [4]. Furthermore, the diagnosis of GIT metastases may be difficult because they often occur many years after the primary cutaneous lesion. It is reported that the DFI until the onset of GIT metastases is 43.8 ± 11.3 months [4]. Metastases confined only to the GIT are rare; in most cases, major organs are already involved at the time of diagnosis. GIT metastases often occur in multiple sites: small bowel (35% to 97%), stomach and duodenum (5% to 50%), and colon (5% to 32%) [5]. There is a significant correlation between their occurrence with the location and nodular type of the primary lesion. Some authors also consider an ulcerated primary lesion as a risk factor for developing GIT metastases. Risk of recurrence is directly correlated to the stage of presentation. In the absence of nodal or distant metastases, stage depends on the thickness and the depth of the primary lesion, determined by two international standardized indexes, the Breslow thickness and the Clark level [6]. A primary lesion with a thickness of between 0.76 and 1.5 mm has up to a 25% chance of developing a regional lymph node recurrence within three years. If the thickness is between 1.5 and 4 mm the risk of nodal recurrence is more than 60% and 15% of these patients develop distant metastases within five years from diagnosis [7]. Moreover, the risk of GIT metastases is higher among patients with a primary lesion classified as Clark level III or above, which is found in 70% to 100% of such patients, although 5% to 24% of patients present with Clark level II and 0% to 6% with Clark level I.

In our patient the primary lesion was found on the left sub-costal region of the trunk. Staging at diagnosis suggested an advanced melanoma, nodular type, exceeding the reticular layer of derma, corresponding to a Clark level IV and a Breslow thickness of 8.3 mm. According to the literature, our patient presented all of the major risk factors for developing GIT metastases: the location of the primary melanoma on the trunk, high Clark level and Breslow thickness, and a nodular type of lesion. Only the gastric metastasis was diagnosed but the presence of other GIT metastases cannot be excluded because small bowel enteroscopy and colonoscopy were not performed. Three types of malignant melanoma features were described at endoscopy: ulcerated melanocytic nodules arising on normal rugae, sub-mucosal masses with ulcerations, and mass lesions with necrosis and melanosis. However, the neoplasia may be completely amelanotic and cytomorphologically variable; in such cases immunohistochemical stains, regardless of the presence or not of melanin pigment, are needed to diagnose malignant melanoma. The most sensitive markers are S100 protein and HMB-45 [8]; in the literature, the sensitivity of S100 varies between 33% and 100% while HMB-45 sensitivity varies between 80% and 97%, with a high specificity (100%) [9,10]. There are other immunohistochemical markers useful in identifying the melanocytic origin of the neoplasia. Melanocytes contain vimentin, an intermediate filament usually expressed in primary and metastatic melanoma cells. However, vimentin positivity can distinguish melanoma from undifferentiated carcinoma, but not from lymphoma or sarcoma [11]. The Melan A protein is a melanocytic differentiation antigen, produced by the MART-1 gene, and it is thought to be specific to melanocytic cells [12]. It was found to be a useful addition to antibody panels when describing cutaneous melanocytic lesions [13]. Tyrosinase is an enzyme involved in the initial stages of melanin biosynthesis in melanocytic and melanoma cells and its hyperexpression has been proposed as a biochemical marker of melanoma [14].

Thus, a broader panel of immuno-markers may be useful in distinguishing between metastases of malignant melanoma and other metastatic malignancies when the lesion is morphologically undifferentiated; Gupta et al. [15], in fact, reported four cases of morphologically undifferentiated melanoma that showed a positivity for HMB45 (two cases), S100 (one case), vimentin (three cases), NKI/C3 (two cases), NKI/Bteb (one case) and CK (three cases). In our patient, the gastric neoplasia was located in the upper third of the stomach, exactly at the passage between fundus and corpus. The gastric neoplasia appeared as a mass covered by fibrin and was amelanotic. These macroscopic findings are similar to the characteristics described in the literature. However, histological findings showed an undifferentiated neoplasm and none of the immunohistochemical stains for melanoma, including S100, HMB-45, Melan A, tyrosinase, CK, CD-34, c-Kit and LCA were able to clarify its origin. The peculiarity of this report is that neither histology nor immunohistochemistry were useful in diagnosing the origin of the lesion, although other authors have reported that tumour markers' loss of expression is not uncommon and this has been experienced at various degrees in other cases of metastatic melanoma [16-18].

Unfortunately, biopsies were only obtained from the stomach lesion; the histological examination of one or more lesions outside of the stomach could have permitted a better characterization of the neoplasia. However, the histological finding of fused cells in the gastric lesion, as featured in the primary melanoma, suggests the diagnosis of GIT metastases. Furthermore, this hypothesis is also supported by the clinical history, the presence of multiple metastases and the occurrence of a neoplasia in the stomach of a patient with all of the major risk factors for developing GIT metastases. Therefore, it is very likely that the undifferentiated gastric neoplasia is a metastasis of the malignant melanoma. Similar cases, characterized by a completely negative immunohistochemistry, have not been described in the literature.

## Conclusion

The present case report shows the difficulty in diagnosing a metastatic melanoma in the GIT, due to its insidious clinical manifestations and morphologic and immunohistochemical variety. This evidence suggests that in a case of melanoma, and during the follow-up and exploration of any gastrointestinal tract disturbances, it is necessary to screen for possible initial or occult metastasis. Therefore, it seems worthwhile to consider metastatic malignant melanoma in the differential diagnosis of undifferentiated neoplasia of the GIT, even in the absence of positive immunohistochemistry.

## Competing interests

The authors declare that they have no competing interests.

## Authors' contributions

FA, PC, AC and AR have made substantial contributions to the conception and design, acquisition of data, or analysis and interpretation of data. FN, PP and DB have been involved in drafting the manuscript or revising it critically and have given final approval of the version to be published.

## Consent

Written informed consent was obtained from the patient's son for publication of this case report and accompanying images. A copy of the written consent is available for review by the Editor-in-Chief of the journal.
